# The Mechanism of Ubiquitination in the Cullin-RING E3 Ligase Machinery: Conformational Control of Substrate Orientation

**DOI:** 10.1371/journal.pcbi.1000527

**Published:** 2009-10-02

**Authors:** Jin Liu, Ruth Nussinov

**Affiliations:** 1 Basic Science Program, SAIC-Frederick, Inc., Center for Cancer Research Nanobiology Program, NCI-Frederick, Frederick, Maryland, United States of America; 2 Sackler Institute of Molecular Medicine, Department of Human Genetics and Molecular Medicine, Sackler School of Medicine, Tel Aviv University, Tel Aviv, Israel; Max-Planck-Institut für Informatik, Germany

## Abstract

In cullin-RING E3 ubiquitin ligases, substrate binding proteins, such as VHL-box, SOCS-box or the F-box proteins, recruit substrates for ubiquitination, accurately positioning and orienting the substrates for ubiquitin transfer. Yet, how the E3 machinery precisely positions the substrate is unknown. Here, we simulated nine substrate binding proteins: Skp2, Fbw7, β-TrCP1, Cdc4, Fbs1, TIR1, pVHL, SOCS2, and SOCS4, in the unbound form and bound to Skp1, ASK1 or Elongin C. All nine proteins have two domains: one binds to the substrate; the other to E3 ligase modules Skp1/ASK1/Elongin C. We discovered that in all cases the flexible inter-domain linker serves as a hinge, rotating the substrate binding domain, optimally and accurately positioning it for ubiquitin transfer. We observed a conserved proline in the linker of all nine proteins. In all cases, the prolines pucker substantially and the pucker is associated with the backbone rotation toward the E2/ubiquitin. We further observed that the linker flexibility could be regulated allosterically by binding events associated with either domain. We conclude that the flexible linker in the substrate binding proteins orients the substrate for the ubiquitin transfer. Our findings provide a mechanism for ubiquitination and polyubiquitination, illustrating that these processes are under conformational control.

## Author Summary

The Ubiquitin-Proteasome System regulates protein degradation via several steps. The cullin-RING E3 ligase machinery is involved in one of these. In this step, ubiquitin is transferred from E2 to the substrate protein, labeling the substrate protein for degradation. However, when E3, E3-substrate and E2-ubiquitin crystal structures are modeled together, the distance between ubiquitinated E2 and the substrate binding site is ∼50–59Å, raising the question how the E3 machinery bridges the distance and orients the substrate for the ubiquitin transfer. We performed explicit solvent simulations for all nine available substrate binding protein complexes in the PDB, with and without the corresponding E3 components to which they are bound. In all of these nine substrate binding proteins, we noticed a flexible linker that rotates the substrate binding domain to a great extent in the same direction, toward the E2-ubiquin. We further noticed that the flexibility is regulated allosterically by binding events associated with either domain. The results suggest that the flexible linker serves as a hinge to rotate the substrate binding domain and to accurately position the substrate for ubiquitination. As such, the simulations suggest an answer to the question of how the machinery operates to orient the substrate for ubiquitination.

## Introduction

**Figure 1:**
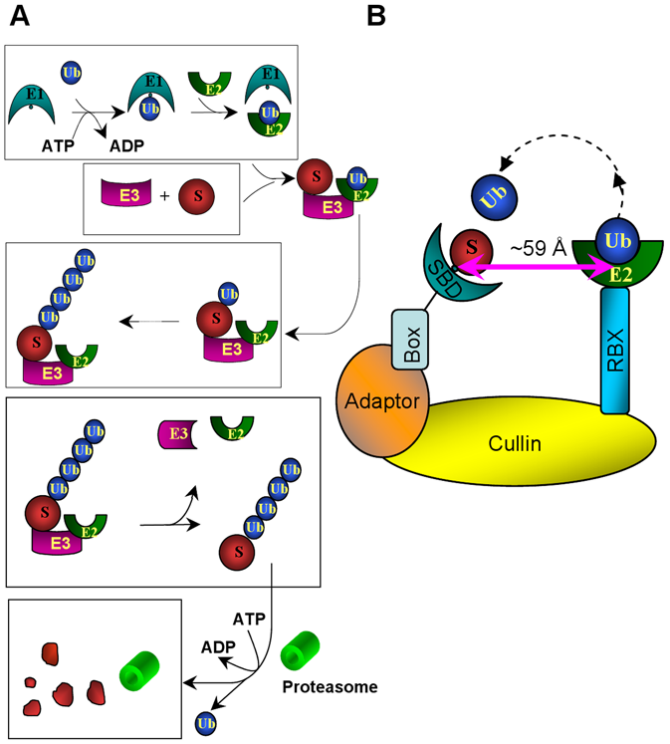
A schematic illustration of the ubiquitin-proteasome system (UPS). (A) Overview of the ubiquitin protein modification pathway. (B) The Ub-E2-E3-substrate machinery.

The Ubiquitin-Proteasome System (UPS) regulates protein degradation in many cellular processes, including signaling, cell-cycle control and development [1]. The ubiquitination of a target protein via the UPS is a highly regulated process, involving several steps (Figure 1A). The 76-amino acid ubiquitin is activated by ubiquitin-activating enzyme E1 with subsequent transfer to the ubiquitin-conjugating enzyme E2. Following formation of the Ub-E2-E3-Substrate complex with ubiquitin ligase E3 and the targeted substrate, ubiquitin is transferred to this substrate. The poly-ubiquitin labeled substrate is recognized and degraded by the proteasome [2].

The ubiquitin system cascade is pyramidal, allowing efficiency and specificity. A single E1 transfers ubiquitin to dozens of E2, which together with hundreds of E3 ubiquitinate thousands of substrates [2],[3]. The way that E3 ligases mediate ubiquitin transfer to substrates divides E3 ligases into two broad categories: HECT E3s and RING/U-box E3s. HECT E3s function in ubiquitin transfer by forming an E3-ubiquitin thioester intermediate, while RING/U-box E3s do not form such intermediates. It is currently believed that RING/U-box E3s bind to the E2-Ub complex and substrate simultaneously, facilitating ubiquitin transfer from E2 to the substrate [4]. There are two sub-categories of RING E3s: Simple RING E3s and multi-module Cullin-RING Ligases (or CRLs). Simple RING E3s have RING-finger E2-binding domain and substrate-binding domain on the same polypeptide; while CRLs consist of four protein modules: RING-Box protein (RBX), which contains the RING domain binding E2; cullin, which is currently thought to constitute a rigid scaffold; adaptor, e.g. Skp1, ASK1 or Elongin C/Elongin B, which connects the substrate-binding protein to the cullin scaffold; and the substrate binding proteins (Figure 1B). Substrate-binding proteins have two domains. One domain has a conserved structure with a three helices “box” motif which binds the adaptor. This domain includes the F-box (e.g. Skp2 [5], Fbw7 [6], β-TrCP1 [7], Cdc4 [8], Fbs1 [9], and TIR1 [10]), VHL-box (e.g. pVHL [11]) and SOCS-box (e.g. the SOCS2 [12] and SOCS4 [13]) families. The other is the substrate binding domain, which could be leucine-rich repeats (Skp2, TIR1), WD-40 repeats (Fbw7, β-TrCP1, or Cdc4), sugar binding domain (Fbs1), β domain (pVHL), or SH2 domain (SOCS2 or SOCS4). All E3 CRL modules form well orchestrated, precise machinery facilitating ubiquitin transfer from E2 to the substrate. It is not clear how this machinery works to ubiquitinate its substrates: One hypothesis posits that the main function of CRLs is to increase the effective concentrations of both substrate and E2-Ub thioester [6],[14]; the other postulates that a box protein contributes to the optimal positioning of the substrate for ubiquitination.

Zheng et al [14] built a model of SCF^Skp2^ (Skp-Cullin-F box protein, where the F-box protein is Skp2) – Rbx - E2 complex by superimposing the Cul1-Rbx1-Skp1-F box on the Skp1-Skp2 complex [5], and docking the UbcH7 E2 onto the Rbx1 RING domain [15]. They observed that even though Skp2 and E2 were on the same side of the SCF complex, the distance between the ubiquitin E2 active site cysteine and the tip of Skp2 is ∼50 Å [14],[16]. Cardozo and Pagano [16] included the p27 substrate complex in the model, presenting a 59 Å distance between the E2 active site and the substrate binding site. This suggests that the orientation of the substrate is crucial in bridging this distance to position the substrate's lysine residue optimally with respect to the ubiquitin's C-terminal to permit the transfer reaction. In the Wu et al. [7] model with the β-Trcp1, a similar 59 Å separation was also measured [16].

Yet, the lack of flexible linkages in the cullin scaffold [14] questions the potential presence of a hinge which would orient the modules in the Ub-E2-E3-Substrate machinery. Further, in addition to the cullin rigidity, based on mutational studies, the linkage between the F-box and the substrate binding domain of the substrate binding protein, and indeed the entire Cul1-Rbx1-Skp1-F box^Skp2^ structure is also believed to be rigid [14]. Recently, however, Duda et al reported a dramatic conformational rearrangement of Rbx1 and Cul5 when bound to ubiquitin-like protein NEDD8. Linker flexibility was observed for Rbx1, which suggests that the E3 ubiquitin ligase machinery can undergo conformational change during ubiquitination [17]. There is also evidence indicating that the linker between the F-box and the substrate binding domain plays an important role in the conformational orientation of the F-box proteins. For example, two crystal forms were identified in the complex of Skp1 and F-box protein Fbs1; whereas Skp1 is well aligned, a rotation angle of 3 degrees between these two crystal forms was observed, suggesting a flexible linker between the F-box and the substrate binding domain [9]. In a second example, the Skp1-Skp2 complex was crystallized, deleting the Skp2 linker and the Skp1 H8 helix to which Skp2 binds; the orientation of Skp2 changed dramatically and the binding of the mutant Skp1-Skp2 was much weaker than the wild type [5]. This implies that the Skp2 linker region could provide a hinge and the binding to Skp1 could trigger the conformational change. Further, there is evidence that mutations in the Cdc4 linker can disrupt the Cdc4 function *in vivo*; this suggests that the Cdc4 linker is critical for the Cdc4 function [8]. A fourth indication that the linker between the two domains in the substrate binding protein could be critical in the ‘correct’ positioning of the substrate for the ubiquitination derives from hydrogen exchange mass spectrometry studies, which showed that the Skp2 substrate-binding domain bound to Cks1 causes a conformational change of the Skp2 linker region [18]. This again implies the intrinsic flexibility of the linkage between the F-box and the substrate binding domain. Moreover, the linker of VHL-box protein pVHL was also observed to be flexible. Sutovsky et al reported that the unbound form pVHL is flexible, but it is stabilized after binding to Elongin C [19]. In addition, previously when simulating pVHL, we observed the linker and interface inter-domain flexibility of pVHL [20].

These observations led us to hypothesize that the flexibility of the inter-domain linkers of substrate-binding proteins *is an intrinsic common feature* for E3 substrate-binding proteins. The linker serves as a hinge to orient the substrate, optimally positioning it for the ubiquitin transfer from E2. This feature could also facilitate the favored orientation during the ubiquitin transfer process in multi-ubiquitin labeling and/or substrate dissociation from the E3 ligase. To test our hypothesis, we performed molecular dynamics simulations for nine substrate binding proteins whose crystal structures are available, including F-box proteins Skp2, Fbw7, β-TrCP1, Cdc4, Fbs1, and TIR1; VHL-box protein pVHL; and SOCS-box proteins SOCS2 and SOCS4, in unbound and bound forms. For all nine simulated proteins, the inter-domain linker regions were flexible in the unbound form, moving away from the E2; while in the bound form, the flexibilities significantly decreased, yet still moving toward E2. We investigated the driving forces and noticed that hydrophobic core formation and charge-charge interaction appear to play an important role. More interestingly, we observed the presence of a conserved proline in the linker region. While there is no cis/trans conformational switch, the prolines demonstrate substantial pucker in the unbound form, and the puckering is significantly decreased in the bound form. We further noticed that the proline is at the hinge and in all nine proteins the puckering is coupled with the backbone conformational change. This observation suggests that this conserved proline has a role in the conformational change in the unbound form and constrains the conformation in the bound form. We propose that intrinsic linker flexibility is a common feature in substrate binding proteins, optimally positioning and orienting the substrate for ubiquitin transfer in the E3 ligase system. Following accurate geometrical positioning for the transfer, the linker flexibility is reduced, moving it further toward optimal conformational orientation.

## Results

### Substrate binding sites overlap

**Figure 2:**
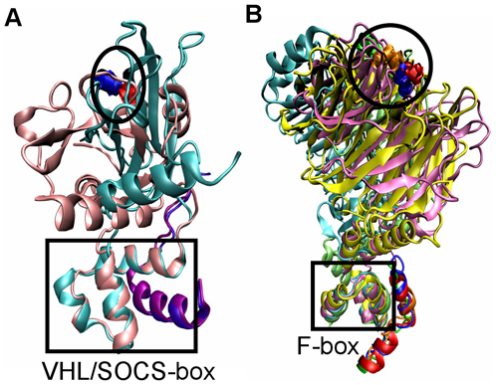
Structure superimposition of substrate-binding proteins. (A) Superposition of Elongin C (purple, violet) and VHL-box/SOCS-box of pVHL (Cyan) (PDB code 1lm8) and SOCS2 (pink) (PDB code 2c9w). The substrate binding domain of pVHL and SOCS2 are different, but their substrate binding sites (blue and red as circled) overlap. (B) Superposition of Skp1 (blue, red, orange or green) and F-box of Skp2 (Cyan) (PDB code 2ast), Fbw7 (pink) (PDB code 2ovq), Cdc4 (yellow) (PDB code 1nex) and Fbs1 (lime) (PDB code 2e31). Their substrate binding sites (blue, red, orange or green) overlap.

VHL-box protein pVHL and SOCS-box proteins SOCS2 and SOCS4 have two domains, the box and the substrate binding domains. The structures of the conserved C-terminal box domains, VHL-box and SOCS-box, respectively, consist of three α helices, H1, H2 and H3; both VHL-box and SOCS-box domains interact with Elongin C. On the other hand, the N-terminal substrate binding domains, β domain for pVHL and SH2 domain for SOCS2 and SOCS4, are very different. Yet, when we superimpose the Elongin C and box domain of pVHL and SOCS2, the distance between the hydroxylation site (Hyp564) of pVHL substrate HIF-1α [11] and the phosphorylated site (pTyr595) of SOCS2 substrate GHR [12] is only 3.0 Å (Figure 2A), suggesting that ubiquitin transfer requires a certain orientation. The proposed phosphorylated site (pTyr1092) of SOCS4 substrate EGFR [13] does not overlap either pVHL's or SOCS2's, which could suggest a different SOCS4 mechanism; alternatively, our simulations (described below) suggest that in the crystal SOCS4 is caught in a local minimum.

Similar to VHL-box and SOCS-box proteins, F-box proteins also have two domains, the F-box and the substrate binding domains. F-box domains are structurally conserved, while the substrate binding domains are not. The substrate binding domains of Skp2 and TIR1 have leucine-rich repeats; Fbs1 has sugar binding domain; whilst those of Fbw7, β-TrCP1 and Cdc4 have a WD-40 substrate binding domain. Nonetheless, superimposing Skp1 and ASK1 of the Skp1/F-box and ASK1/F-box of the six F-box complexes as an anchor, leads to an interesting result: four of these proteins, Skp2, Fbw7, Cdc4 and Fbs1 overlap the sites that the substrates bind: pThr187 of Skp2 substrate p27 [21], pThr380 of Fbw7 substrate cyclin E [6], pThr4 of Cdc4 substrate CPD [8], and high-mannose oligosaccharide attached Asn34 of Fbs1 substrate RNaseB [9], respectively (Figure 2B). The distances among these sites are less than 3 Å. The exceptions are β-TrCP1 and TIR1, whose substrate binding sites are 10–15 Å away from the other four proteins, which may suggest a different mechanism for ubiquitin transfer; alternatively, again, our simulations (below) suggest crystals trapped in local minima.

### Conformational flexibility after binding to Skp1, ASK1 or Elongin C

**Figure 3:**
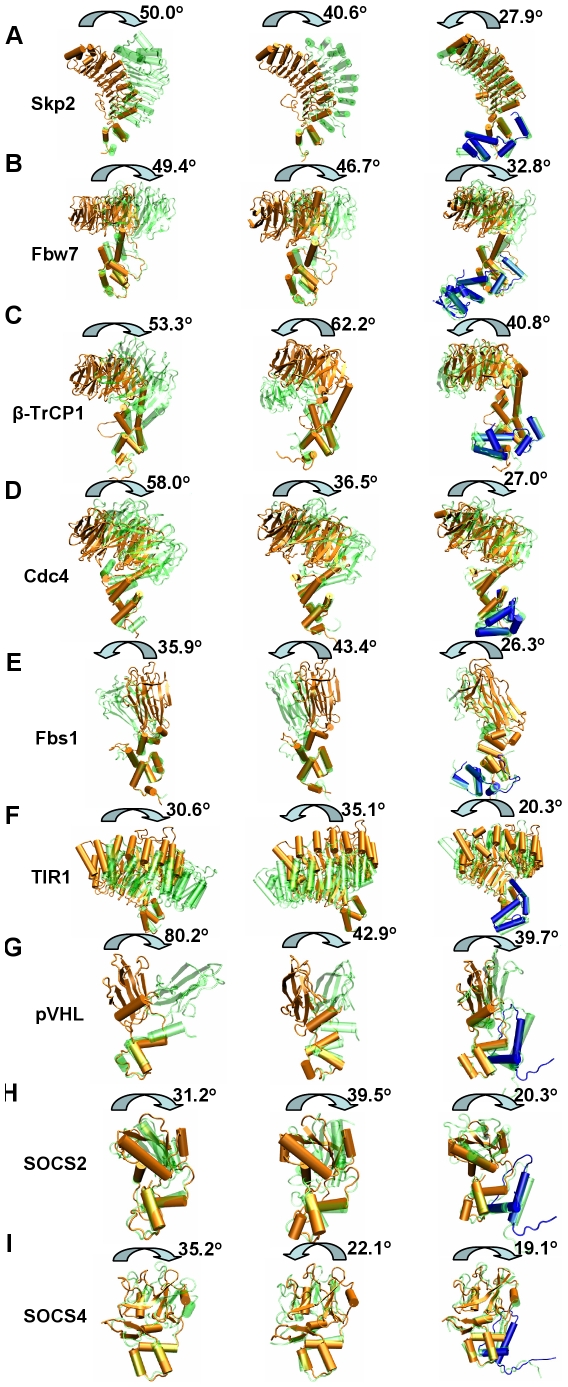
Comparison of snapshots from the simulations. (A) Skp2; (B) Fbw7; (C) β-TrCP1; (D) Cdc4; (E) Fbs1; (F) TIR1; (G) pVHL; (H) SOCS2; (I) SOCS4. The unbound form trajectory 1(left), trajectory 2 (middle) and bound form (right) comparison is shown in each figure. Structural snapshots of the F-box, VHL-box and SOCS-box domains are superimposed. The snapshots are taken at 0 ns (orange for substrate binding proteins and blue for adaptor Skp1, ASK1 or Elongin C) and maximum rotation angle (green). The rotation angles of the substrate binding domain are shown.

To our knowledge, complexed crystal structures are available for five Skp1-binding F-box proteins, Skp2, Fbw7, β-TrCP1, Cdc4, and Fbs1; one ASK1-binding F-box protein TIR1, and three Elongin C- binding proteins, VHL-box protein pVHL, and two SOCS-box proteins, SOCS2 and SOCS4. Molecular dynamics simulations were performed for all nine proteins in the unbound and bound (Skp2, Fbw7, β-TrCP1, Cdc4 and Fbs1 to Skp1; TIR1 to ASK1; pVHL, SOCS2 and SOCS4 to Elongin C) forms. To decrease the chances of the results dependence on the starting conditions, repeated simulations were performed for all the unbound forms. The rotation angles for both unbound (including two independent simulations) and bound forms are shown in Figure 3, Figure S1, S2 and Table S1. The results shown in all other figures and tables are from first simulation of the unbound and bound forms.

**Figure 4:**
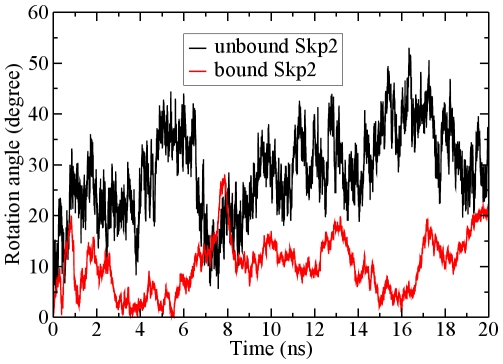
Rotation angles of Skp2 unbound (black) and bound (red) forms during the simulations.

For the unbound form, when the conserved box domains are superimposed, the substrate binding domains rotate up to 30–80 degrees with respect to their corresponding box domains in the 20 nanosecond simulations. Figure 3 depicts the superimposed structures at 0 ns and the snapshots with maximum rotation angles. All nine proteins have more obvious rotations in the unbound form as compared to the bound in both trajectories. Figure 4 plots the rotation angles of Skp2 for the unbound (first trajectory) and bound forms. The unbound Skp2 has larger rotation angles than the bound form. The rotation angles for the other proteins are shown in Figure S1 (for the Skp1/ASK1-binding) and Figure S2 (for the Elongin C-binding) proteins. Among these nine proteins, the VHL-box protein pVHL rotation in the first simulation is the largest, with a maximum of 80 degrees, and an average of 37.5. The rotation of F-box proteins Skp2, Fbw7, β-TrCP1 and Cdc4 fluctuate more, with a maximum between 36–62 degrees, and the average between 15–30 degrees for both trials. The SOCS-box and F-box proteins Fbs1 and TIR1 rotations are the smallest, with the maximum angles around 22–45 degrees and the average between 9–18 degrees. For SOCS4 and Fbs1, the rotation in the first trajectory is barely noticeable at 300K, but increases significantly in either second trajectory or when we raised the simulation temperature to 340K, suggesting that they had to climb out of a local minimum and overcome a barrier. All of these proteins have similar rotation axes, which extend through the inter-domain interface.

In the bound form simulations the rotations have significantly decreased with the substrate binding domain still moving further toward E2-ubiquitin. Compared to the unbound form, the maximum and the average rotation angles are much smaller: the maximum angles of the F-box proteins Skp2, Fbw7, β-TrCP1 and Cdc4 are 27.9, 32.8, 40.8 and 27.0 degrees, respectively (Figure S1 and Table S1), decreasing by 10–31 degrees, comparing to both simulations of the unbound form. The average rotation angles also decrease to 18.3, 6.1 and 12.7 degrees for Skp2, Fbw7 and Cdc4, respectively. The only exception is β-TrCP1, whose mean bound conformation rotation angle increases by 3.9 degrees compared to the first unbound trajectory, but decreases by 0.4 degrees comparing to second unbound trajectory, again suggesting that the crystal structure of this complex may be at a local minimum. The maximum and average rotation angles of pVHL bound form are 40 and 22 degrees, decreasing by 40 and 16 degrees, respectively, compared to the first trajectory of the unbound form. The maximum rotation of the second unbound trajectory of pVHL is not as large as the first trajectory, probably due to the starting conditions of the simulation, but still 3 degrees larger than the bound form. As for SOCS-box proteins SOCS2 and SOCS4, and F-box proteins Fbs1 and TIR1, the maximum rotation angles decrease by 3–24 degrees, and the average rotation angles also decrease (Table S1). There are no significant differences between the rotation angles for bound SOCS4 and Fbs1 at 340K and 300K (Figure S1, S2). The standard deviations for rotation angles changes during the simulation are included in Table S1.

### Driving force for the rotation

We searched for the driving forces for the conformational change. The inter-domain interface structures of F-box proteins are quite different than those of the VHL-box and SOCS-box proteins. The three helix bundle and interface of F-box proteins form a cavity. In the unbound form, they tend to form a hydrophobic core consisting of H2 of the F-box and an α helix (H4, H5, H7, H6, H6 or H4 for Skp2, Fbw7, β-TrCP1, Cdc4, Fbs1, or TIR1, respectively) next to the substrate binding domain. Hydrophobic core formation can assist in driving the conformational change in F-box proteins. The distance changes between the hydrophobic residues of F-box proteins during the simulations are shown in Figure S1. The distance lines roughly match the rotation angle graphs. When the distance between the hydrophobic residues decreases, the rotation angle increases. When these proteins are bound to Skp1 or ASK1, however, the cavity is filled by Skp1 H8 or ASK1 H7, thus the rotations of the bound form are much less than those of the unbound. In pVHL, the interface has two charged residues at the inter-domain interface: Arg82 and Arg161. The distance (Figure S2) and the rotation angle graphs of the pVHL unbound form show that the rotation angles increase with an increase in the charged residues distances. Thus, charge-charge repulsion could also play a role in driving the pVHL conformational change. After binding to Elongin C, both Arg82 and Arg161 interact with Elongin C Glu35 and the pVHL inter-domain interface is stabilized.

For the SOCS-box proteins SOCS2 and SOCS4, charge-charge interactions could also play a role in inter-domain rotation. SOCS2 has two positively charged residues at the interface, Arg41 and Arg168. During the simulations, these two charged residues separated from 4.6 Å to more than 20 Å (Figure S2). Even though there are no direct interactions between Elongin C and these two charged residues, it appears that Arg41 and Arg168 are stabilized allosterically by binding to Elongin C. For SOCS4, the attraction between Glu336 and Lys427 could also have a role in driving the inter-domain rotation. This attraction is weakened allosterically after SOCS4 binding to Elongin C; SOCS4 is stabilized by binding to Elongin C.

### Conserved proline at the linker can assist in the control of the conformation change

**Figure 5:**
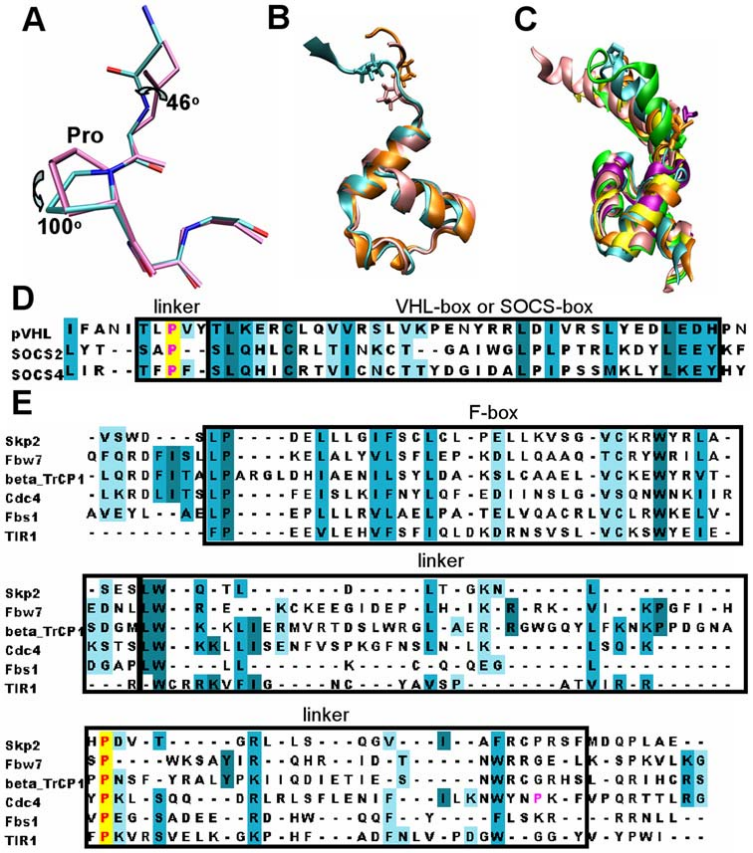
Conserved prolines in the linker region. (A) Skp2 proline puckering up and down is coupled with backbone conformational change. Two snapshots from simulations with prolines puckering up and down were superimposed and the backbone rotations are shown. (B) Superposition of pVHL (Cyan), SOCS2 (pink), and SOCS4 (orange) box domain with prolines at the linker. (C) Superposition of Skp2 (Cyan), Fbw7 (pink), β-TrCP1 (orange), Cdc4 (yellow), Fbs1 (purple) and TIR1 (green) with prolines at the linker. (D) Sequence alignment of pVHL, SOCS2 and SOCS4. (E) Sequence alignment of Skp2, Fbw7, β-TrCP1, Cdc4, Fbs1 and TIR1.

The rotation hinges are in the linker region for all nine proteins. For TIR1, the LRR1 serves as its linker region. The VHL-box and SOCS-box proteins have only one hinge region in the short linker between the two domains, while the F-box proteins have two hinge regions: the first is in the short turn next to the F-box; the second in the α helix next to the substrate binding domain. Specifically, the second hinge is at the beginning of the α helix of Skp2, Fbw7 and β-TrCP1, Fbs1, but at the end of the α helix of Cdc4 and TIR1. Sequence analysis of the hinge region (Figure 5D–E) shows one common feature: all nine proteins have a proline residue. The superimposed VHL-box and SOCS-box protein structures with the conserved prolines are shown in Figure 5B; superimposed F-box proteins with prolines at the hinge in Figure 5C. Note that the prolines are at the beginning of Skp2, Fbw7 and β-TrCP1, Fbs1, but at the other end of Cdc4 and TIR1, just where the hinge is. Alignments were further performed for sequences obtained from BLAST searches on all non-redundant protein sequences from peptide sequence databases, including GenBank, RefSeq, PDB, SWISS-PROT, PIR and PRF, for each of these nine protein families. The proline conservation percentage is from 52% to 100% for these nine families. Details are shown in Figure S6 and Table S2. The prolines in the linkers of the substrate-binding proteins do not display a “proline door” with cis/trans- conformational change, as in well-documented proline-gated ion channels [22]. Instead, the prolines stay in a trans- form during the simulations; however, the conserved prolines do pucker tremendously in the unbound form, and the puckering is significantly decreased in the bound form, as shown in Table 1. In the unbound form, the proline pucker ratio is much smaller than that in the bound form, which suggests that in the bound form, the down proline position is dominant and that the proline is more likely to pucker in the unbound form. Similar to the Ho et al observation that the puckering in the proline ring is coupled to the backbone conformation change [23], we noticed that the nearby backbone conformation changes with the proline puckering in all nine proteins. Figure 5A superimposes two snapshots taken from the Skp2 unbound form simulations with the proline in the up and down states, showing the backbone change. This suggests that proline plays a role in the control of the conformational change: in the unbound form, the backbone change coupled with the proline puckering promotes the rotation of the substrate binding domain; while in the bound form the dominant down position of the proline constrains the conformation with the substrate binding domain fluctuating toward its optimal position.

**Table 1:** The ratio of down/up proline pucker in the unbound versus bound simulations.

		Skp2	Fbw7	β-TrCP1	Cdc4	Fbs1	TIR1	pVHL	SOCS2	SOCS4
Proline down/up	unbound	3.1	1.7	1.6	2.8	3.2	9.2	1.7	3.2	4.2
	bound	5.6	3.8	4.5	7.7	3.8	14.2	4.9	4.2	10.3

The numbers of the prolines puckering down or up are counted in the snapshots.

### Substrate binding sites are correlated with Skp1, ASK1 or Elongin C binding sites

Covariance maps were generated for the nine proteins for the unbound and bound form simulations. Covariance maps are useful in identifying regions whose motions are correlated or anti-correlated and as such can assist in discovering allosterically-related residues [20]. Figure S4A shows the covariance maps for Skp2. The linker region is positively correlated with both the F-box and the substrate binding domains in both the unbound and bound forms. However, the correlation in the bound is much stronger than that in the unbound form, which suggests that after binding to Skp1, the Skp2 linker movement is more coupled to the substrate binding domain allosterically. The covariance maps for the other proteins are in Figure S4, S5. For both the F-box and VHL-box proteins, the correlations between the linker and the two domains become much stronger for the bound than they were in the unbound form. For the SOCS box proteins, both the unbound and bound forms have strong correlations between the linker and the two domains. The strong positive correlations observed in the bound form for all nine proteins imply rigidification, constraining a specific, ubiquitination more favored orientation [14].

## Discussion

Here we investigate a crucial mechanistic detail of the E3 ligase system: in order for ubiquitin to be efficiently transferred to its cognate substrates, the substrates have to be precisely spatially positioned and oriented with respect to the E2-ubiquitin (Figure 1B). Yet, current evidence suggests that the E3 machinery is likely to be rigid [14], with the active sites of the E2 and the substrate at a distance as far as 50 to 59 Å, which raises the question how the E3 machinery accomplishes this task. Clearly, the substrate binding domain should orient such that the distance to the E2-ubiquitin could be bridged. We used atomic scale molecular dynamics simulations to look into this E3 mechanistic enigma. Even though the E3 machinery movement could be on a micro- to millisecond time scale, the connection between the local atomic fluctuations on nanosecond time scale and the global conformational transitions on microsecond time scale has been well established [24]. Here, we performed simulations on nine available complexes of the E3 substrate binding proteins, F-box proteins Skp2, Fbw7, β-TrCP1, Cdc4, Fbs1 and TIR1, VHL-box protein pVHL, and SOCS-box protein SOCS2 and SOCS4. All nine have two domains, a structurally conserved box domain bound to the adjoining E3 ligase modules, and a substrate binding domain bound to the substrate. The two domains are connected by flexible linkers. The unbound state simulations clearly showed that if we take the box domain as the anchor, the linker will act as the hinge and rotate the substrate binding domain for a maximum of 30–80 degrees in the 20 nanosecond simulations. To reduce the possibility that the observed motion reflects the starting conditions, we performed a second set simulation for all unbound states and still observed large rotations for all simulated proteins. When bound to other E3 modules, the linker flexibility decreases; however, the substrate binding domain still moves further toward E2. In general, increased protein stabilities following binding are expected locally at the binding sites, not necessarily affecting the motions of other domains. Since most of the linkers and substrate binding domains are not included in the adaptor binding sites, the binding of the box domain to the adaptor stabilizes the substrate binding domain allosterically.

**Figure 6:**
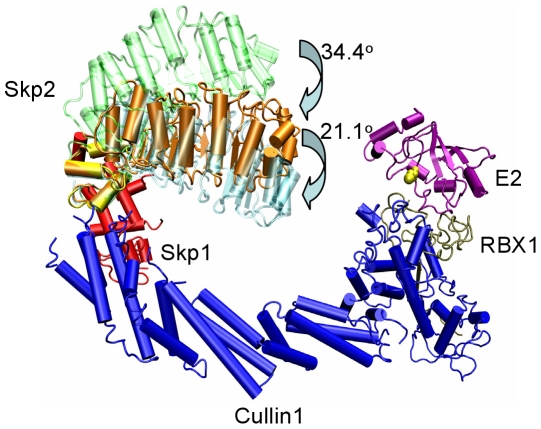
Model of the E2-Rbx1-Cul1-Skp1-Skp2 complex. E2 (purple, PDB code 1fbv) is docked to Rbx1(gray)-Cul1(blue)-Skp1(red)-Skp2 F-box (yellow) complex (PDB code 1ldk). Skp2 snapshots at 0 ns (orange) and 20 ns (green) for unbound form and 20 ns (cyan) for bound form are superimposed on the crystal structure using the F-box domain as the pivot.

In a recent conformational study of ubiquitin [25], the NMR ensemble covered the structural heterogeneity of 46 ubiquitin crystal structures, most of which are complexes of ubiquitin with other proteins, invoking conformational selection of ubiquitin conformers rather than an induced-fit mechanism [26]. This observation supports the earlier proposition of conformational selection with consequent population shifts [27]–[32] in ubiquitination [33]. Thus, all ubiquitin conformers are present in solution; the ones which are most favored for a given target selectively bind. These concepts of conformational selection and the consequent re-distributions of protein conformational ensembles in allostery [34]–[36] are increasingly being accepted [33],[37]. Recent literature already presents a broad range of conformational selection and population shift examples, mostly made possible by remarkable recent advances in NMR. These include protein-protein, protein-ligand and DNA/RNA. Here, due to the linker flexibility, the substrate binding proteins cannot be crystallized in their free states; consequently, we are not able to start simulations from such states and search the visited states for bound-like conformers. However, the simulations of the unbound forms of the substrate binding proteins indicate that they exist in an ensemble of conformations. Thus, we propose that a higher energy bound-like conformer is favored to bind Skp1, ASK1 or Elongin C through conformational selection. Binding would stabilize the conformer, with population shift propagating this binding reaction, leading to an observable conformational change with the substrate-binding domain in a more favorable position for ubiquitin transfer. Induced fit would optimize the substrate binding protein-adaptor interaction. The F-box, pVHL-box and SOCS-box binding sites are strongly positively correlated with their respective linkers (Figure S4, S5). When we superimpose the snapshots of the unbound and bound Skp2 at 0 ns and 20 ns, respectively, with the crystal structures of the E2 and E3 complexes [14],[15],[33] (Figure 6), it is clear that the complex structure has a much more favorable position for ubiquitin transfer. However, the complex structure is not rigid either; the bound forms also exist in a conformational ensemble. During the 20 ns simulation, the rotations, although to smaller extents comparing to the unbound forms, still take place also in the bound forms, which helps the movement of substrate binding domain to a more favored position for the ubiquitination (and poly-ubiquitination) of the substrate. Superimposing snapshots of Fbw7, β-TrCP1 Cdc4, Fbs1 and TIR1 (Figure S3) gives similar results.

Further, superposition of the two Elongin C complexes, with pVHL and with SOCS2, indicates that the respective substrate binding sites overlap even though their substrate binding domains are structurally dissimilar; while in SOCS4 the position is different. Yet, the necessity to raise the temperature to 340 degrees in the SOCS4 simulations to observe the hinge motion and re-orientation (Figure S2) suggests that crystallization trapped a conformer in a local minimum. Similarly, superposition of Skp1 in the complex-form again indicates an overlap of the substrate binding sites of Skp2, Fbw7, Cdc4 and Fbs1, with the exception of β-TrCP1 and TIR1. However, β-TrCP1 has a more flexible bound form than other F-box proteins (Figure S1), again raising the possibility that crystallization trapped another conformer, more populated under those conditions. The small rotation angles of TIR1 in both unbound and bound forms also suggest a crystal-trapped form. Fbw7 and Cdc4 have similar substrate binding domains, which are completely different from either Skp2 or Fbs1. Fbw7 has two substrate binding sites, whereas the others have one. Surprisingly, these different binding modes could make the substrate binding sites overlap. While the spatial juxtaposition of the ubiquitin-receiving lysine(s) and the substrate binding sites vary, these sites can communicate allosterically, triggering further linker movement which facilitates ubiquitin transfer. Common substrate binding sites do not imply fixed spatial location; rather, these positions could be conformationally-selected, favored for ubiquitin transfer.

Hydrophobic core formation for F-box proteins and charge-charge interactions for VHL-box and SOCS-box proteins could be the driving forces for the conformational change of the linker region. We further noticed a conserved proline in the linkers of all nine proteins. Conformational analysis indicated a large difference in the proline pucker between the bound and unbound simulations. Proline ring puckering has been coupled to the backbone conformation change [23], which is also observed here (Figure 5A); as such it assists in orienting the linker toward the E2. In all nine proteins, we observe a strong positive correlation between the proline and the substrate binding domain in the covariance maps of the bound simulations (Figure S5, S6). Proline substitution in the Rbx1 linker was recently reported to restrict conformational change in the E3 complex [17]. It will be interesting to test the catalytic efficiency of proline substitution or deletion in the substrate binding protein linker.

Covariance maps also show strong correlations between the linker and the two domains. The coupled motion between the substrate binding domain and the linker implies that substrate binding can allosterically affect the linker conformation. These results are consistent with recent hydrogen exchange mass spectrometry showing that Skp2 substrate-binding domain binding to Cks1 causes a conformation change of the Skp2 linker [18]. We also noticed that the correlation between the linker and the substrate binding domain is stronger following the box binding to the adaptor, which explains the experimental results that the substrate domain binding to Csk1 further stabilizes the Skp1/Skp2 binding. The strong correlation between the linker and other parts of the substrate binding proteins could further assist in ubiquitin transfer by allosterically re-orienting the linker in poly-ubiquitin elongation.

**Figure 7:**
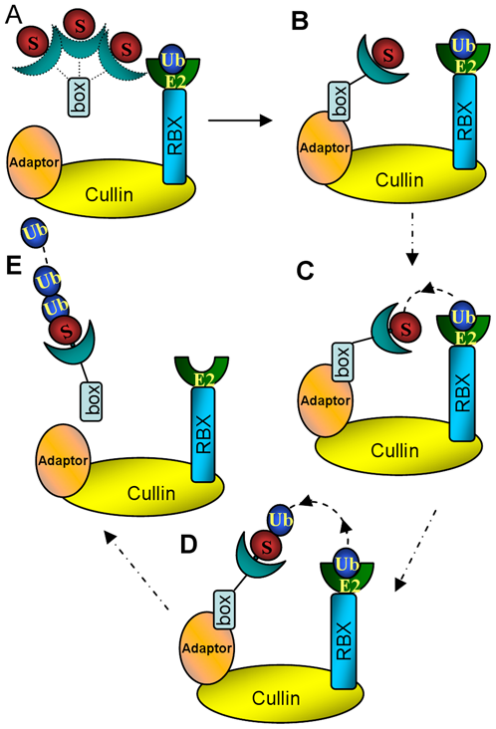
A scheme of the proposed pathway. (A) Prior to binding to other E3 modules, the linker is flexible. (B) In the favored E3-bound conformation the substrate binding domain is rotated on the linker to the optimal position. (C) The strong correlations in the motions between the linker and the substrate binding domain in the bound state, suggest allosteric effects with the linker further rotating the substrate binding domain following substrate binding for optimal ubiquitin transfer position. (D) The linker rotates to facilitate additional ubiquitin transfer. (E) The linker rotation facilitates the poly-ubiquitin-labeled substrate dissociation from the E3 ligase.

To conclude, Figure 7 describes a possible scenario for substrate recruitment. In the unbound state, substrate binding proteins are in a conformational ensemble with a range of angles between the two domains. The E3 adaptor, such as Skp1, ASK1, or Elongin C, selects [25],[27],[28],[33] binding-ready conformations; these further orient toward E2 to facilitate ubiquitin transfer. Following the first ubiquitin transfer, the flexible linker can re-adjust for subsequent ubiquitinations. The strong correlations in the motions of the linker and the substrate binding domain suggest that the substrate binding domain flexibility, which is correlated with linker flexibility, has the potential to weaken its interaction with substrate thus facilitate dissociation of the ubiquitin-labeled substrate from the E3 ligase. The linker is intrinsically flexible, and could be regulated allosterically [38]. Searching for allosteric sites could provide a new strategy for drug discovery targeting the ubiquitin system.

## Methods

### System setup

The starting structures of the unbound and bound forms of nine proteins, Skp2, Fbw7, β-TrCP1, Cdc4, Fbs1, TIR1, pVHL, SOCS2, and SOCS4 were obtained from crystal structures (PDB codes: 2ast, 2ovq, 1p22, 1nex, 2e31, 2p1q, 1lm8, 2c9w, and 2ziv). The starting structures of the bound forms were abstracted with substrate removed, except SOCS2 and SOCS4, whose substrates were not in crystal structure, whereas the starting structures of the unbound form were created by removing all the binding partners, including the adaptor and the substrate, if applicable. For the pVHL bound to Elongin C, only Elongin C residues 58–112 were used since coordinates were unavailable for the eight-residue gap between residues 49 and 58, and the solved Elongin C structure for residues 17 to 49 was far away from the pVHL-Elongin C binding site. The starting structure of SOCS2/SOCS4 -Elongin C complexes was generated with similar Elongin C truncation. All models were solvated in a TIP3P water box with a minimum distance of 10 Å from the edge of the box to any protein atom. The system charges were neutralized by adding chloride or sodium ions.

### Simulation protocol

Molecular dynamics (MD) simulations were performed with CHARMM 27 [39] force field using the NAMD program [40]. Even though normal mode calculations are powerful in obtaining domain rotations, they are not able to provide atomic-level details as driving forces and interactions and preferred side chain states. Therefore here we chose to use explicit solvent MD simulations despite the computational costs. To eliminate residual unfavorable interactions between the solvent and the protein, the solvated systems were first minimized for 3000 steps with the protein restrained followed by another 3000 steps of minimization with all atoms allowed to move. Then the systems were heated from 0 K to 300K in 100 ps constraining protein backbone atoms to allow the relaxation of solvent molecules. The systems were then equilibrated for 100 ps with constrained protein backbone atoms followed by 500 ps equilibrium run without any constraints. Production simulations were performed for 20 ns with the NPT ensemble at 300K and room pressure. For SOCS4 and Fbs1, simulations at 340K were also performed. Temperature and pressure were controlled using Langevin thermostat and Nose-Hoover Langevin piston barostat as implemented in NAMD. The short range interactions employed a switch function with 12 Å cutoff and 10 Å switch distance, and the long range electrostatic interactions were calculated with particle mesh Ewald summation. During the production simulations, the time step was 2 fs, with a SHAKE constraint on all bonds containing hydrogen atoms.

Structural alignments and figure rendering were performed by VMD. The angle rotations at 0 and 20 ns were calculated by DynDom [41] and the angle rotation analysis during the simulation were performed using Hingefind [42]. The sequence alignments search were performed by BLAST [43].

## Supporting Information

Figure S1 Angle rotation graphs of unbound trajectory 1 (black), trajectory 2 (blue) and bound (red) forms for (A) Skp2, (C) Fbw7, (E) β-TrCP1, (G) Cdc4, (I) Fbs1, and (K) TIR1. The graphs of changes in the distances between hydrophobic residues from box domain and linker are shown for the unbound form trajectory1 of (B) Skp2, (D) Fbw7, (F) β-TrCP1, (H) Cdc4, (J) Fbs1 and (L) TIR1.
(1.82 MB PDF)

Figure S2 Angle rotation graphs of unbound trajectory 1 (black), trajectory 2 (blue) and bound (red) form for (A) pVHL, (C) SOCS2, (E) SOCS4. The graphs of distance changes between the charged residues at the inter-domain interface are shown for the unbound form trajectory1 of (B) pVHL, (D) SOCS2, (F) SOCS4.
(0.98 MB PDF)

Figure S3 Models of the E2-Rbx1-Cul1-Skp1 complex superimposed with (A) Fbw7 (B) β-TrCP1 (C) Cdc4(D) Fbs1 and (E) TIR1. E2 (purple) is docked to Rbx1(gray)-Cul1(blue)-Skp1(red)-Skp2 F-box (yellow) complex (PDB code 1LDK). Snapshots of (A) Fbw7 (B) β-TrCP1 (C) Cdc4 (D) Fbs1 and (E) TIR1 at 0 ns (orange) and 20 ns (green) for unbound form and 20 ns (cyan) for bound form are superimposed with Skp2 F-box domain.
(1.04 MB PDF)

Figure S4 Covariance maps of (i) unbound and (ii) bound form of (A) Skp2 (B) Fbs1 (C) TIR1 (D) Fbw7 (E) β-TrCP1 and (F) Cdc4. The position of the prolineis marked. The more red, the stronger the positive correlation; the more blue the stronger the negative (anti-) correlation. The bar provides the scale.
(2.50 MB PDF)

Figure S5 Covariance maps of (i) unbound and (ii) bound form of (A) pVHL(B) SOCS2 and (C) SOCS4. The position of the prolineis marked. The more red, the stronger the positive correlation; the more blue the stronger the negative (anti-) correlation. The bar provides the scale.
(0.72 MB PDF)

Figure S6 Sequence alignment of (A) VHL-box, SOCS-box and (B) F-box proteins.
(0.10 MB PDF)

Table S1 Rotation angles (degrees) for nineproteins.
(0.12 MB PDF)

Table S2 Sequence analysis of the conserved prolines. The sequences of box-domain and linker region for each protein were used as query sequences to search for matching sequences using BLAST.
(0.09 MB PDF)
