# Supplementary material for: The Mechanism of Ubiquitination in the Cullin-RING E3 Ligase Machinery: Conformational Control of Substrate Orientation
Source: PLoS Comput Biol. 2009 Oct 2;5(10):e1000527. doi: 10.1371/journal.pcbi.1000527 (PMC2741574; doi:10.1371/journal.pcbi.1000527)
Supplement: Figure S1 — Angle rotation graphs of unbound trajectory 1 (black), trajectory 2 (blue) and bound (red) forms for (A) Skp2, (C) Fbw7, (E) β-TrCP1, (G) Cdc4, (I) Fbs1, and (K) TIR1. The graphs of changes in the distances between hydrophobic residues from box domain and linker are shown for the unbound form trajectory1 of (B) Skp2, (D) Fbw7, (F) β-TrCP1, (H) Cdc4, (J) Fbs1 and (L) TIR1. (1.82 MB PDF) [file pcbi.1000527.s001.pdf]

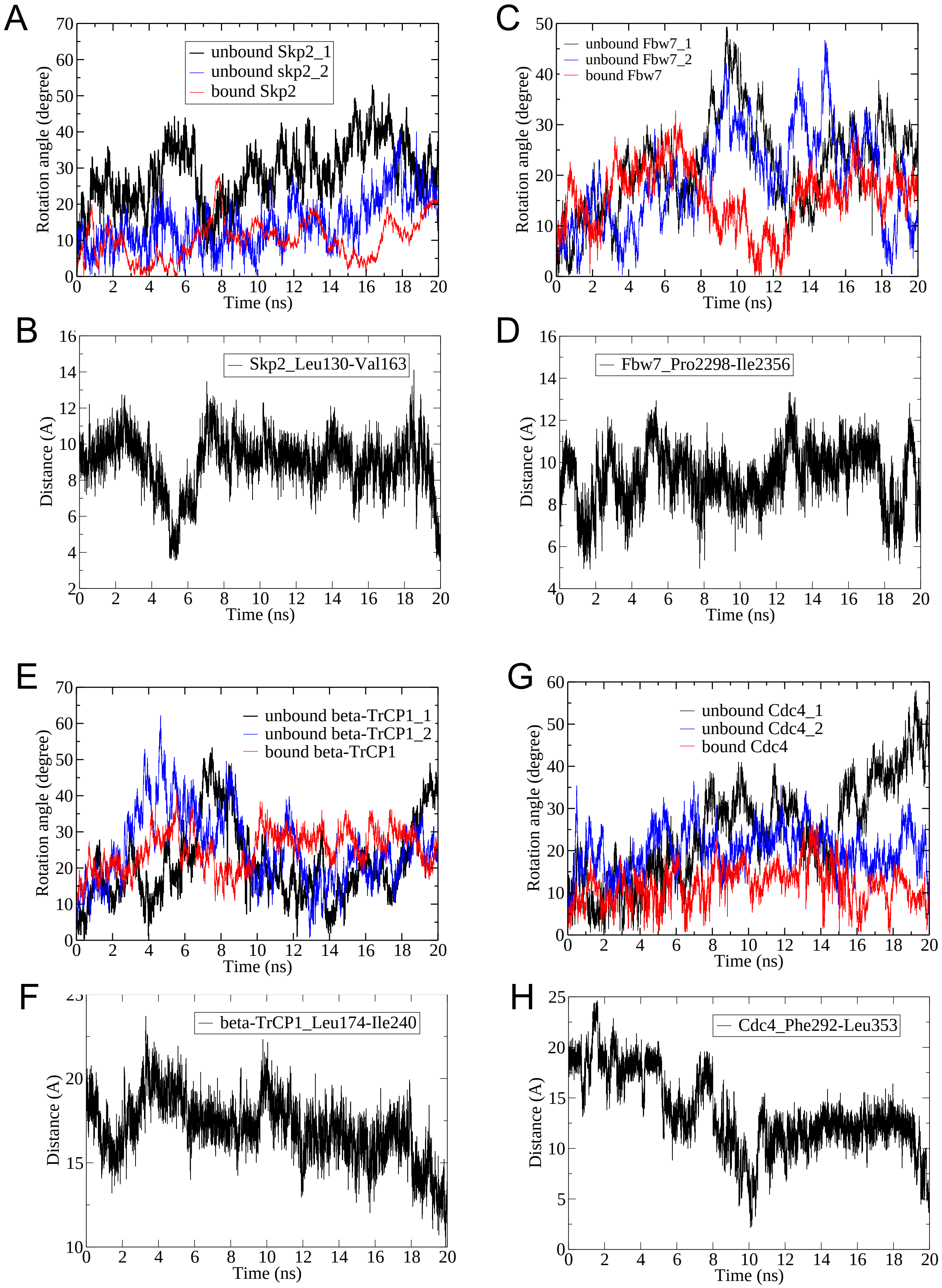
A
unbound Skp2_1
unbound skp2_2
bound Skp2
Rotation angle (degree)
Time (ns)
B
Skp2_Leu130-Val163
Distance (A)
Time (ns)
C
unbound Fbw7_1
unbound Fbw7_2
bound Fbw7
Rotation angle (degree)
Time (ns)
D
Fbw7_Pro2298-Ile2356
Distance (A)
Time (ns)
E
unbound beta-TrCP1_1
unbound beta-TrCP1_2
bound beta-TrCP1
Rotation angle (degree)
Time (ns)
F
beta-TrCP1_Leu174-Ile240
Distance (A)
Time (ns)
G
unbound Cdc4_1
unbound Cdc4_2
bound Cdc4
Rotation angle (degree)
Time (ns)
H
Cdc4_Phe292-Leu353
Distance (A)
Time (ns)

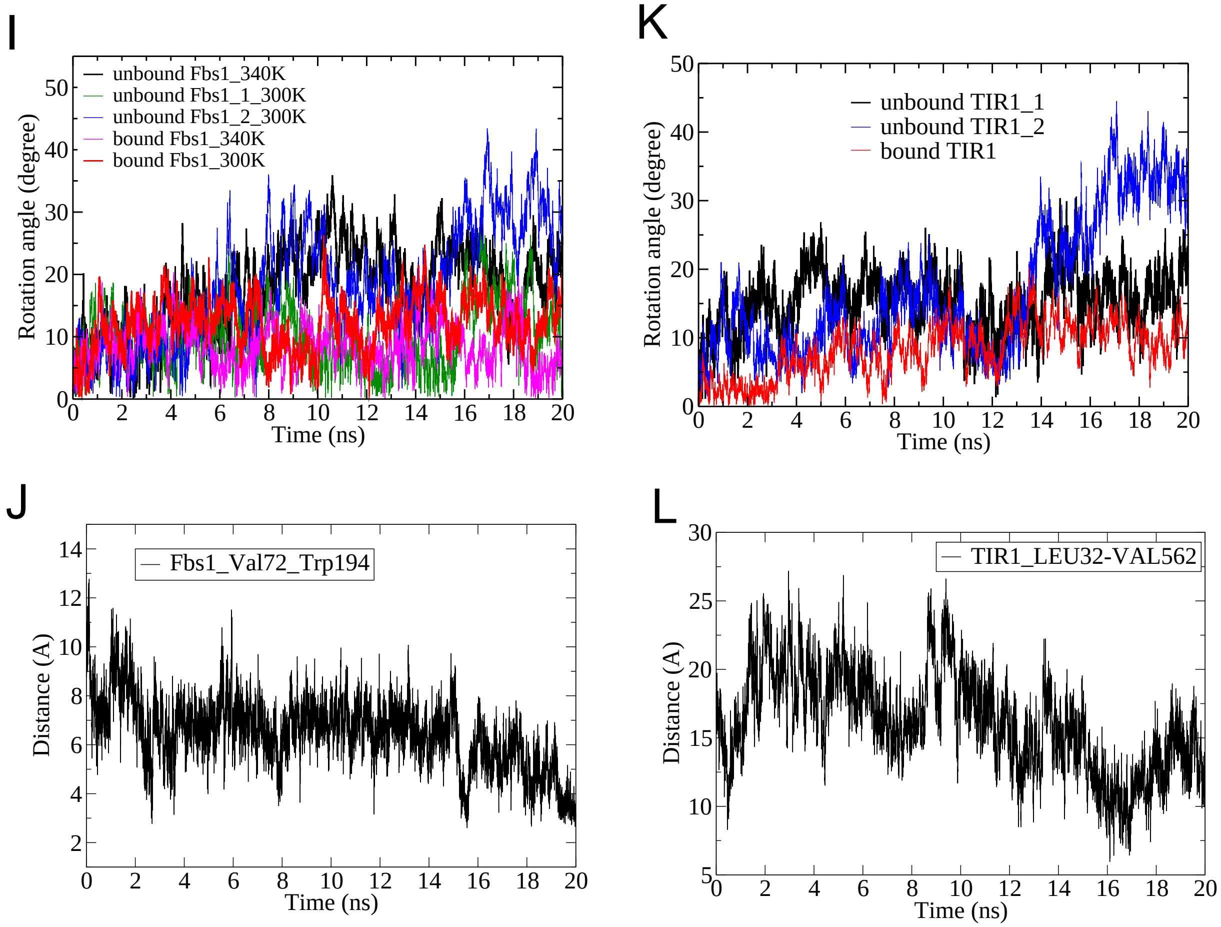

Figure S1. Angle rotation graphs of unbound trajectory 1 (black), trajectory 2 (blue) and bound (red) forms for (A) Skp2, (C) Fbw7, (E) β-TrCP1, (G) Cdc4, (I) Fbs1, and (K) TIR1. The graphs of changes in the distances between hydrophobic residues from box domain and linker are shown for the unbound form trajectory 1 of (B) Skp2, (D) Fbw7, (F) β-TrCP1, (H) Cdc4, (J) Fbs1 and (L) TIR1.
