# Supplementary material for: The Mechanism of Ubiquitination in the Cullin-RING E3 Ligase Machinery: Conformational Control of Substrate Orientation
Source: PLoS Comput Biol. 2009 Oct 2;5(10):e1000527. doi: 10.1371/journal.pcbi.1000527 (PMC2741574; doi:10.1371/journal.pcbi.1000527)
Supplement: Figure S2 — Angle rotation graphs of unbound trajectory 1 (black), trajectory 2 (blue) and bound (red) form for (A) pVHL, (C) SOCS2, (E) SOCS4. The graphs of distance changes between the charged residues at the inter-domain interface are shown for the unbound form trajectory1 of (B) pVHL, (D) SOCS2, (F) SOCS4. (0.98 MB PDF) [file pcbi.1000527.s002.pdf]

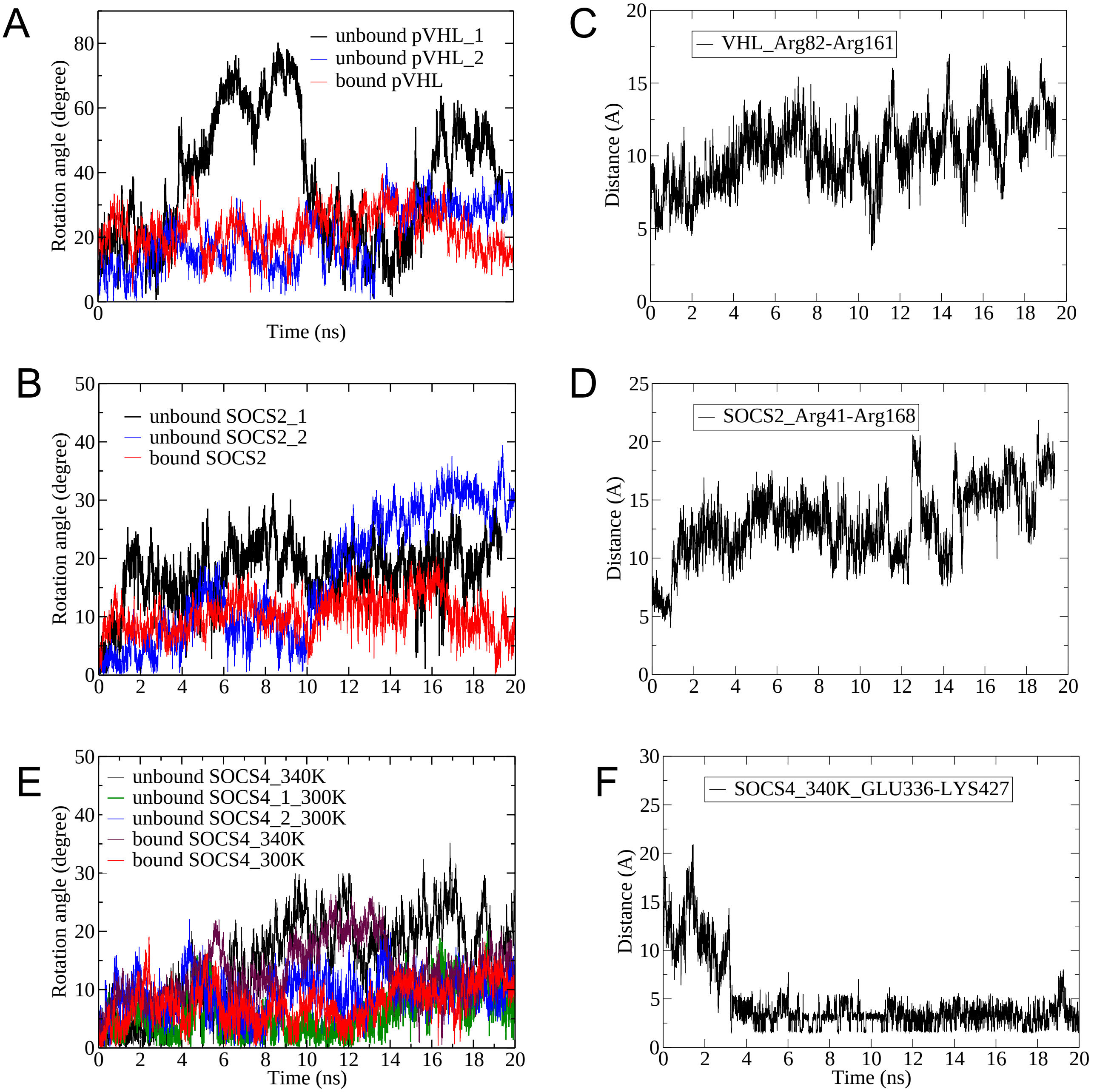

Figure S2. Angle rotation graphs of unbound trajectory 1 (black), trajectory 2 (blue) and bound (red) form for (A) pVHL, (C) SOCS2, (E) SOCS4. The graphs of distance changes between the charged residues at the inter-domain interface are shown for the unbound form trajectory 1 of (B) pVHL, (D) SOCS2, (F) SOCS4.
