# Supplementary material for: The Mechanism of Ubiquitination in the Cullin-RING E3 Ligase Machinery: Conformational Control of Substrate Orientation
Source: PLoS Comput Biol. 2009 Oct 2;5(10):e1000527. doi: 10.1371/journal.pcbi.1000527 (PMC2741574; doi:10.1371/journal.pcbi.1000527)
Supplement: Figure S3 — Models of the E2-Rbx1-Cul1-Skp1 complex superimposed with (A) Fbw7 (B) β-TrCP1 (C) Cdc4(D) Fbs1 and (E) TIR1. E2 (purple) is docked to Rbx1(gray)-Cul1(blue)-Skp1(red)-Skp2 F-box (yellow) complex (PDB code 1LDK). Snapshots of (A) Fbw7 (B) β-TrCP1 (C) Cdc4 (D) Fbs1 and (E) TIR1 at 0 ns (orange) and 20 ns (green) for unbound form and 20 ns (cyan) for bound form are superimposed with Skp2 F-box domain. (1.04 MB PDF) [file pcbi.1000527.s003.pdf]

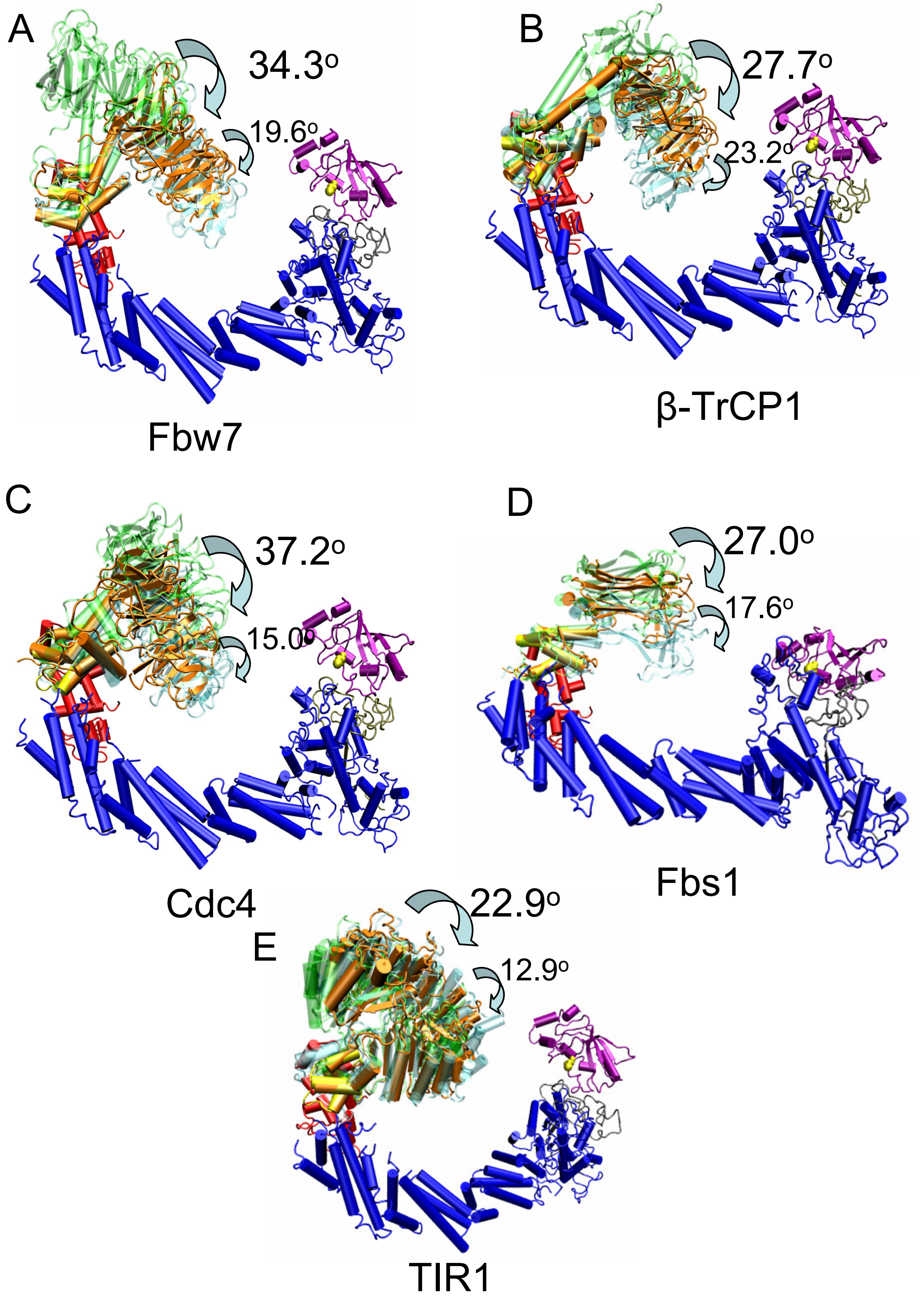

Figure S3. Models of the E2-Rbx1-Cul1-Skp1 complex superimposed with (A) Fbw7 (B) β-TrCP1 (C) Cdc4 (D) Fbs1 and (E) TIR1. E2 (purple) is docked to Rbx1(gray)-Cul1(blue)-Skp1(red)-Skp2 F-box (yellow) complex (PDB code 1LDK). Snapshots of (A) Fbw7 (B) β-TrCP1 (C) Cdc4 (D) Fbs1 and (E) TIR1 at 0 ns (orange) and 20ns (green) for unbound form and 20ns (cyan) for bound form are superimposed with Skp2 F-box domain.
