# Supplementary material for: The Mechanism of Ubiquitination in the Cullin-RING E3 Ligase Machinery: Conformational Control of Substrate Orientation
Source: PLoS Comput Biol. 2009 Oct 2;5(10):e1000527. doi: 10.1371/journal.pcbi.1000527 (PMC2741574; doi:10.1371/journal.pcbi.1000527)
Supplement: Figure S4 — Covariance maps of (i) unbound and (ii) bound form of (A) Skp2 (B) Fbs1 (C) TIR1 (D) Fbw7 (E) β-TrCP1 and (F) Cdc4. The position of the prolineis marked. The more red, the stronger the positive correlation; the more blue the stronger the negative (anti-) correlation. The bar provides the scale. (2.50 MB PDF) [file pcbi.1000527.s004.pdf]

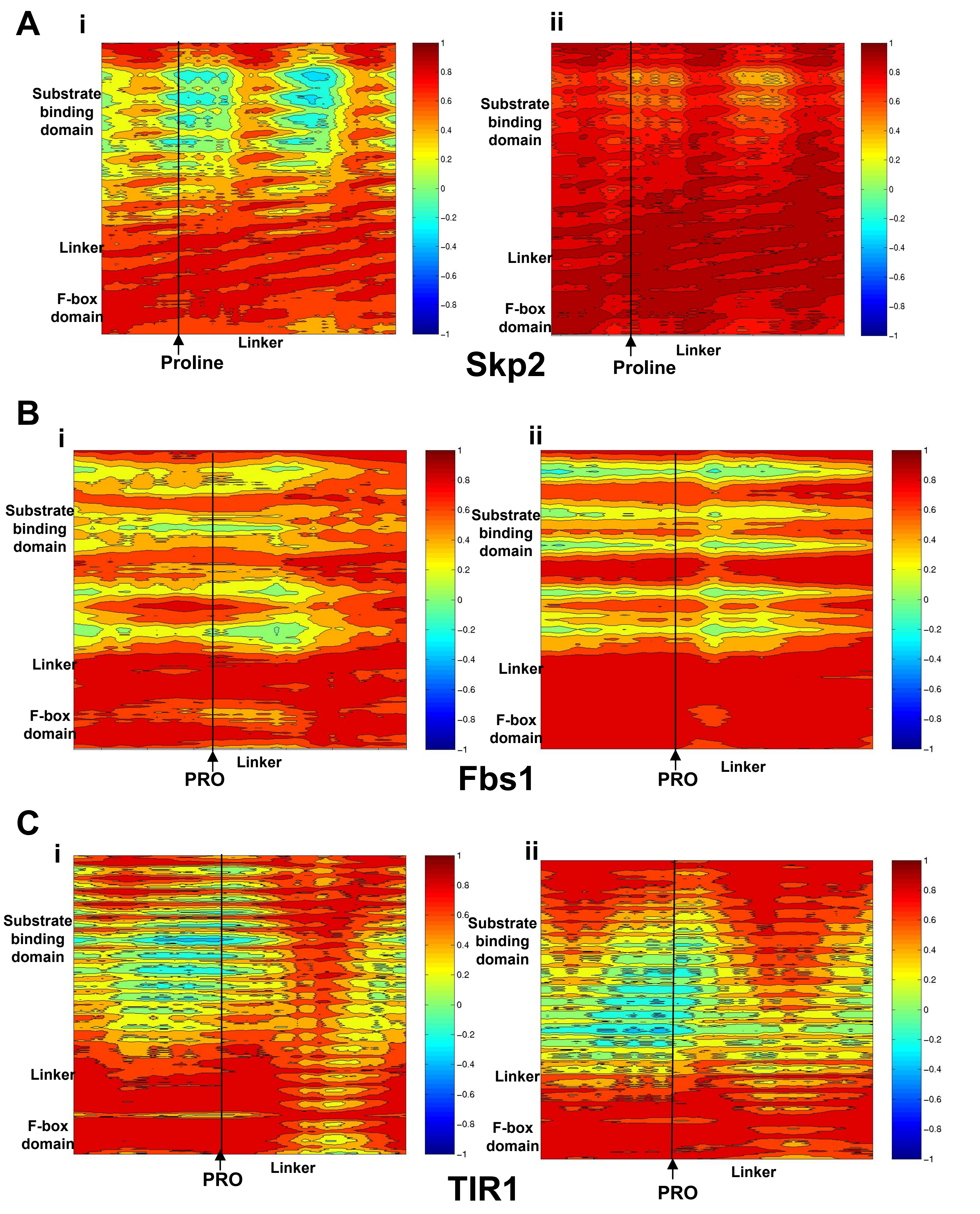
A
i
ii
Substrate binding domain
Linker
F-box domain
Proline
Linker
Skp2
B
Fbs1
PRO
C
TIR1
1
0.8
0.6
0.4
0.2
0
−0.2
−0.4
−0.6
−0.8
−1

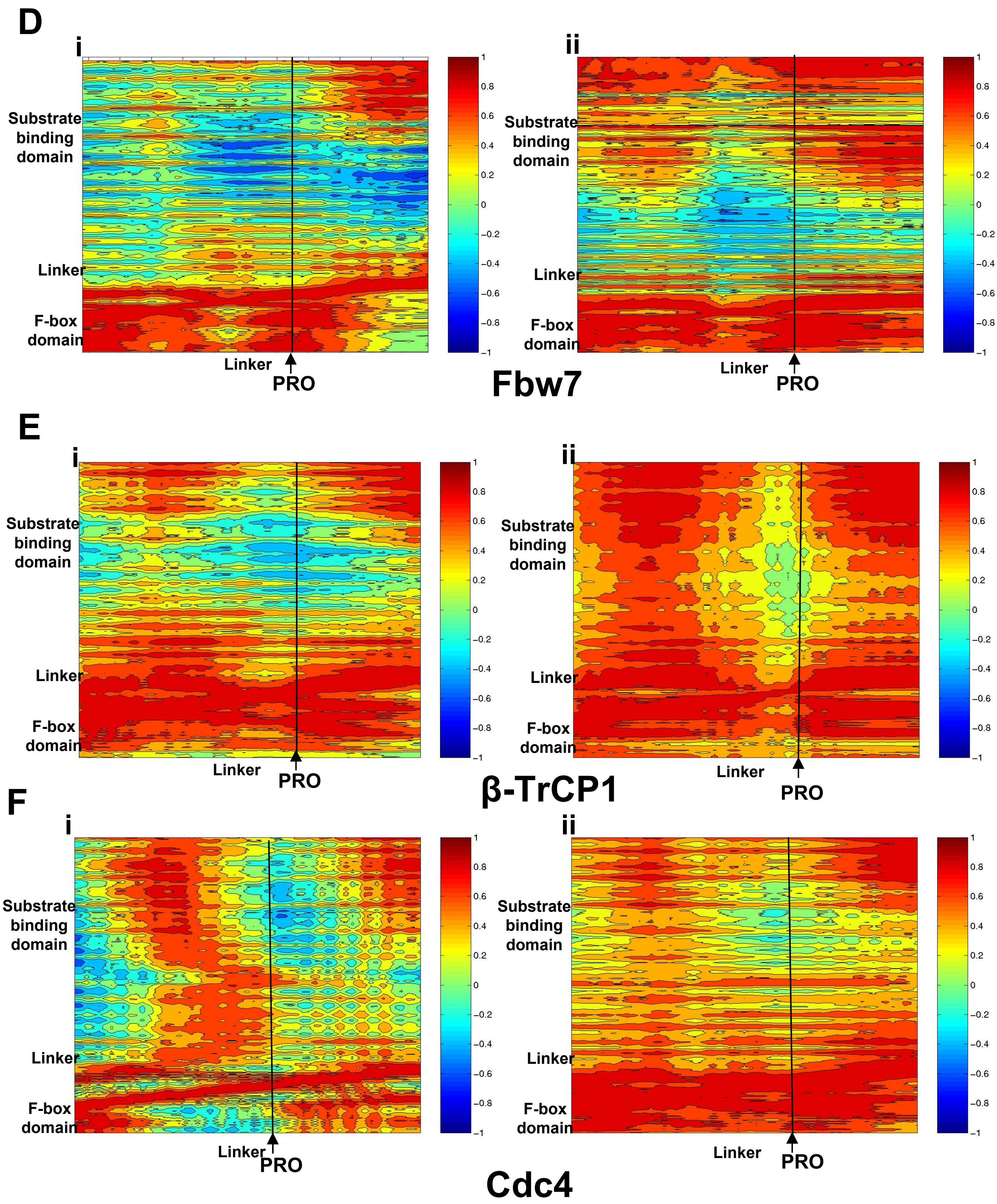

Figure S4. Covariance maps of (i) unbound and (ii) bound form of (A) Skp2 (B) Fbs1 (C) TIR1 (D) Fbw7 (E) β-TrCP1 and (F) Cdc4. The position of the proline is marked. The more red, the stronger the positive correlation; the more blue the stronger the negative (anti-) correlation. The bar provides the scale.
