# Supplementary material for: The Mechanism of Ubiquitination in the Cullin-RING E3 Ligase Machinery: Conformational Control of Substrate Orientation
Source: PLoS Comput Biol. 2009 Oct 2;5(10):e1000527. doi: 10.1371/journal.pcbi.1000527 (PMC2741574; doi:10.1371/journal.pcbi.1000527)
Supplement: Figure S5 — Covariance maps of (i) unbound and (ii) bound form of (A) pVHL(B) SOCS2 and (C) SOCS4. The position of the prolineis marked. The more red, the stronger the positive correlation; the more blue the stronger the negative (anti-) correlation. The bar provides the scale. (0.72 MB PDF) [file pcbi.1000527.s005.pdf]

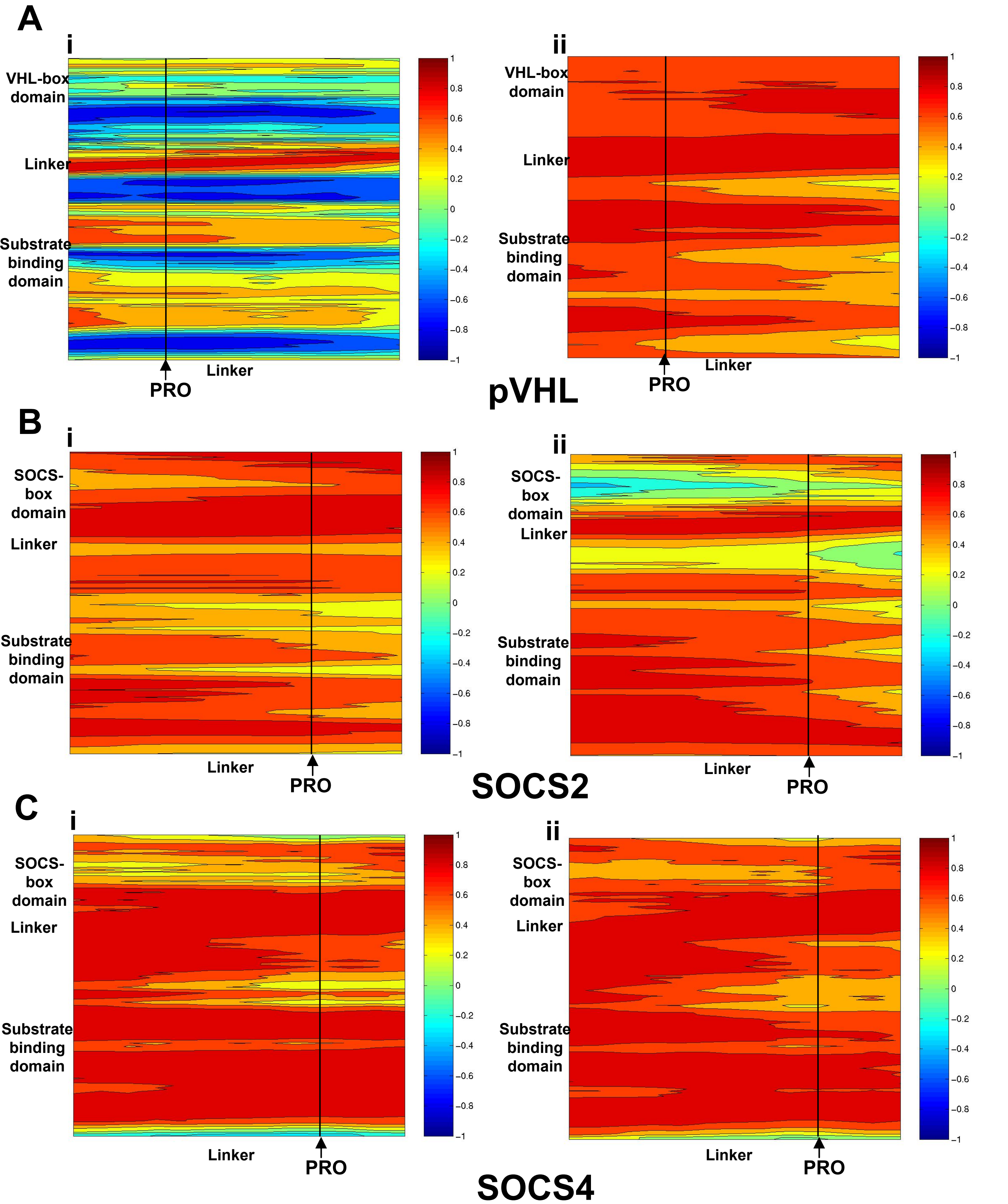

Figure S5. Covariance maps of (i) unbound and (ii) bound form of (A) pVHL (B) SOCS2 and (C) SOCS4. The position of the proline is marked. The more red, the stronger the positive correlation; the more blue the stronger the negative (anti-) correlation. The bar provides the scale.
