# Supplementary material for: The Mechanism of Ubiquitination in the Cullin-RING E3 Ligase Machinery: Conformational Control of Substrate Orientation
Source: PLoS Comput Biol. 2009 Oct 2;5(10):e1000527. doi: 10.1371/journal.pcbi.1000527 (PMC2741574; doi:10.1371/journal.pcbi.1000527)
Supplement: Figure S6 — Sequence alignment of (A) VHL-box, SOCS-box and (B) F-box proteins. (0.10 MB PDF) [file pcbi.1000527.s006.pdf]

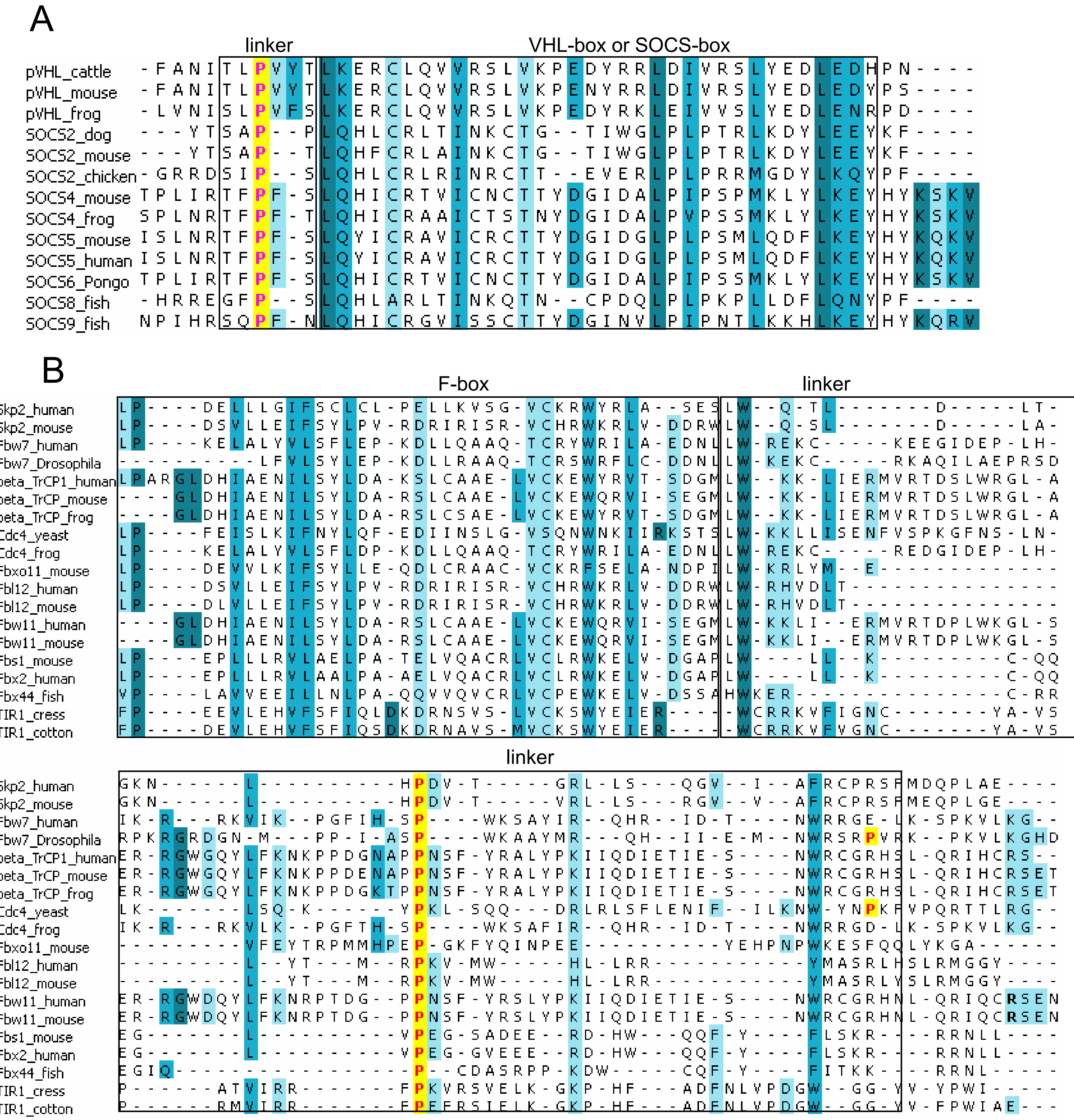

Figure S6. Sequence alignment of (A) VHL-box, SOCS-box and (B) F-box proteins.
