# Supplementary material for: The Mechanism of Ubiquitination in the Cullin-RING E3 Ligase Machinery: Conformational Control of Substrate Orientation
Source: PLoS Comput Biol. 2009 Oct 2;5(10):e1000527. doi: 10.1371/journal.pcbi.1000527 (PMC2741574; doi:10.1371/journal.pcbi.1000527)
Supplement: Table S1 — Rotation angles (degrees) for nineproteins. (0.12 MB PDF) [file pcbi.1000527.s007.pdf]

Table S1. Rotation angles (degrees) for nine proteins.

		Skp2	Fbw7	β-TrCP1	Cdc4	Fbs1	TIR1	pVHL	SOCS2	SOCS4
**Unbound Trajectory 1**	**Max**	53.0	49.4	53.3	58.0	35.9	30.6	80.2	31.2	35.2
	**Mean**	28.5	21.9	21.2	24.5	17.7	15.3	37.5	17.9	15.0
	**Stdev**	8.5	9.0	10.6	11.7	6.8	4.3	19.5	5.0	7.6
**Unbound Trajectory 2**	**Max**	40.6	46.7	62.2	36.5	43.4	44.5	42.9	39.5	22.1
	**Mean**	14.5	19.4	25.5	20.2	18.2	16.7	19.4	16.6	9.3
	**Stdev**	6.9	9.4	10.0	5.1	9.1	9.9	8.8	10.6	3.4
**bound**	**Max**	27.9	32.8	40.8	27.0	26.3	20.3	39.7	20.3	19.1
	**Mean**	10.2	15.8	25.1	11.8	12.1	8.3	22.0	10.3	7.7
	**Stdev**	5.4	5.9	5.4	4.5	4.1	3.9	5.9	3.2	3.3
