# Supplementary material for: The Mechanism of Ubiquitination in the Cullin-RING E3 Ligase Machinery: Conformational Control of Substrate Orientation
Source: PLoS Comput Biol. 2009 Oct 2;5(10):e1000527. doi: 10.1371/journal.pcbi.1000527 (PMC2741574; doi:10.1371/journal.pcbi.1000527)
Supplement: Table S2 — Sequence analysis of the conserved prolines. The sequences of box-domain and linker region for each protein were used as query sequences to search for matching sequences using BLAST. (0.09 MB PDF) [file pcbi.1000527.s008.pdf]

Table S2. Sequence analysis of the conserved prolines. The sequences of box-domain and linker region for each protein were used as query sequences to search for matching sequences using BLAST.

	Skp2	Fbw7	β-TrCP1	Cdc4	Fbs1	TIR1	pVHL	SOCS2	SOCS4
Percent Pro conservation[a]	63%	74%	95%	100%	52%	78%	100%	78%	64%
No. of aligned protein sequences[b]	83	83	95	7	70	64	17	80	33

[a]The percentages for the aligned proteins with conserved proline residue at the linker region.
[b]Total numbers of proteins assigned to the respective families and matched by BLAST. Predicted, hypothetical, and unknown proteins were excluded.
